# HDL Glycoprotein Composition and Site-Specific Glycosylation Differentiates Between Clinical Groups and Affects IL-6 Secretion in *Lipopolysaccharide*-Stimulated Monocytes

**DOI:** 10.1038/srep43728

**Published:** 2017-03-13

**Authors:** Sridevi Krishnan, Michiko Shimoda, Romina Sacchi, Muchena J. Kailemia, Guillaume Luxardi, George A. Kaysen, Atul N. Parikh, Viviane N. Ngassam, Kirsten Johansen, Glenn M. Chertow, Barbara Grimes, Jennifer T. Smilowitz, Emanual Maverakis, Carlito B. Lebrilla, Angela M. Zivkovic

**Affiliations:** 1 Department of Nutrition, University of California, Davis, CA 95616, USA; 2 Department of Dermatology, University of California, Davis School of Medicine, Sacramento, CA 95816, USA; 3 Department of Chemistry, University of California, Davis, CA 95616, USA; 4 Biochemistry and Molecular Medicine; Internal Medicine, University of California Davis School of Medicine, Davis CA 95616, USA; 5 Department of Biomedical Engineering, University of California, Davis, CA 95616 USA; 6 Department of Medicine, University of California San Francisco, School of Medicine, San Francisco, CA 94121, USA; 7 Department of Medicine, Nephrology, Stanford School of Medicine, Stanford CA 94305, USA; 8 Department of Epidemiology and Biostatistics, University of California, San Francisco CA, USA; 9 Department of Food Science & Technology, University of California, Davis, CA 95616, USA.

## Abstract

The goal of this pilot study was to determine whether HDL glycoprotein composition affects HDL’s immunomodulatory function. HDL were purified from healthy controls (n = 13), subjects with metabolic syndrome (MetS) (n = 13), and diabetic hemodialysis (HD) patients (n = 24). Concentrations of HDL-bound serum amyloid A (SAA), *lipopolysaccharide* binding protein (LBP), apolipoprotein A-I (ApoA-I), apolipoprotein C-III (ApoC-III), α-1-antitrypsin (A1AT), and α-2-HS-glycoprotein (A2HSG); and the site-specific glycovariations of ApoC-III, A1AT, and A2HSG were measured. Secretion of interleukin 6 (IL-6) in *lipopolysaccharide-*stimulated monocytes was used as a prototypical assay of HDL’s immunomodulatory capacity. HDL from HD patients were enriched in SAA, LBP, ApoC-III, di-sialylated ApoC-III (ApoC-III_2_) and desialylated A2HSG. HDL that increased IL-6 secretion were enriched in ApoC-III, di-sialylated glycans at multiple A1AT glycosylation sites and desialylated A2HSG, and depleted in mono-sialylated ApoC-III (ApoC-III_1_). Subgroup analysis on HD patients who experienced an infectious hospitalization event within 60 days (HD+) (n = 12), vs. those with no event (HD−) (n = 12) showed that HDL from HD+ patients were enriched in SAA but had lower levels of sialylation across glycoproteins. Our results demonstrate that HDL glycoprotein composition, including the site-specific glycosylation, differentiate between clinical groups, correlate with HDL’s immunomodulatory capacity, and may be predictive of HDL’s ability to protect from infection.

High density lipoproteins (HDL) are primarily known for their atheroprotective functions, particularly reverse cholesterol transport, reducing oxidation of LDL, and protecting the endothelial wall^1^. In the past ten years, additional functions of HDL have been discovered, including those related to inflammation^2^ and immune function^3^. A recent analysis of multiple genome-wide association studies of inflammatory diseases comprising a total of over 200,000 individuals found multiple shared risk loci between HDL and immune pathways^4^.

HDL has three known pathways by which it interacts with the immune system. (1) By various mechanisms HDL can protect against septic shock^5^. Specifically, it can bind and neutralize lipopolysaccharide (LPS) produced by gram-negative bacteria for subsequent transfer to and degradation by the liver^6^,^7^. It can also protect against septic shock by transferring LPS to Low Density Lipoproteins (LDL)^8^. (2) HDL particles can deplete cholesterol from immune cells, thereby reducing lipid raft-associated signalling^9^. (3) HDL particles can have a direct anti-inflammatory effect on immune cells via the transcriptional down-regulation of cytokines^10^. Importantly, for each of these studies HDL was prepared from pooled serum samples; thus, the existence of patient-to-patient variation in the immunomodulatory properties of HDL is currently unknown. Given the wide inter-individual compositional variation of HDL, identifying the components of HDL that correlate with its effect on immune response is critical.

Our current understanding of the inter-individual variability in HDL’s immunomodulatory effects is rudimentary and somewhat contradictory. For example, investigators have demonstrated that the HDL-associated proteins, apolipoprotein A-I (ApoA-I)^11^, serum amyloid A (SAA)^12^, and ApoC-III^13^ have opposing immunomodulatory properties. ApoA-I suppresses cytokine release in a variety of cell types^10^,^11^ whereas SAA stimulates the release of cytokines and chemotaxis^12^. Also, it has been demonstrated that HDL loses its anti-inflammatory and anti-oxidant capacity in the setting of the acute phase response^14^,^15^. Likewise, it has been shown that HDL’s anti-inflammatory capacity is reduced in metabolic diseases including type 2 diabetes and MetS (reviewed in ref. 16). Given these results, we hypothesize that the net immunomodulatory capacity of an individual’s HDL will be dependent on their specific HDL composition.

However, correlating HDL composition with function is a daunting task because the highly complex HDL proteome has over 100 proteins, many of which are associated with cholesterol transport and lipid metabolism. How these proteins are also associated with the acute phase response, immunity, and inflammation is an area of active investigation^17^,^18^,^19^. Another dimension of complexity is added by the fact that most of the HDL-associated proteins are glycosylated and the individual structures of the decorating glycans will likely alter the immunomodulatory properties of the proteins^20^. To focus on the individual glycovariants of the HDL-associated proteins, we have previously developed a method to characterize the HDL glycoproteome^21^, and identified the importance of both sialylation and fucosylation in distinguishing among individuals who have coronary artery disease from those who do not^22^. Another group has reported a similar finding. In HDL from patients with MetS both the amount of ApoC-III and SAA, and the glycosylation of ApoC-III, were altered compared to healthy controls^23^. Thus, it is becoming increasingly apparent that there is differential expression of the HDL glycoproteome in health and disease; however, little is known about how glycan alterations affect HDL function.

The primary objectives of this study were to determine whether HDL glycoprotein composition: (1) differentiates between clinical groups, (2) modifies the effect of HDL on response phenotype in monocytes stimulated with *lipopolysaccharide* (LPS), and (3) is associated with risk for infectious hospitalization in a high-risk group. Using our in-house developed technology, a detailed analysis of HDL composition was conducted on each sample including the concentrations of the HDL bound proteins; ApoC-III, α-1-antitrypsin (A1AT), and α-2-HS-glycoprotein (A2HSG), the quantification of the site-specific glycovariations of these proteins, as well as the concentrations of ApoA-I, serum amyloid A (SAA), LPS binding protein (LBP), cholesterol, and measurement of HDL particle size by dynamic light scattering. Our results demonstrate that HDL composition, including the site-specific glycosylation, differentiates between clinical groups, correlates with HDL’s ability to modulate the LPS-induced monocyte cytokine response, and may be predictive of HDL’s ability to protect against infection.

## Methods

### Sample Collection

Serum samples from a total of 50 subjects who had participated in three previously conducted human studies were analyzed for this study. Five healthy control subjects who were part of a training run for the larger study, and 13 participants diagnosed with MetS who participated in a human study to assess the effects of high fat meals on postprandial inflammation, were randomly selected for this project. Both the healthy and MetS subjects had to meet a set of inclusion criteria, including not currently taking any medications and not having experienced any recent illnesses. MetS was characterized as having three out of the following five metabolic traits as defined by the American Heart Association: waist circumference >40 inches for men and 35 inches for women, fasting plasma triglyceride (TG) ≥ 150 mg/dL, fasting plasma HDL cholesterol (HDL-C) <40 mg/dL for men and <50 mg/dL for women, blood pressure ≥130/85 mmHg, and fasting glucose ≥100 mg/dL^24^. The Institutional Review Board of the University of California at Davis approved this study protocol, and all participants gave written informed consent prior to starting the study, as well as consent for the use of their biological specimens and anonymized data in follow-up studies. All methods of this study were carried out in accordance with approved guidelines. The study was registered at clinicaltrials.gov under NCT01811329. Seven additional healthy control subjects who participated in a methods development study donated blood samples through a study that was approved by the Institutional Review Board of the University of California at Davis. Subjects provided written informed consent and the study followed all approved guidelines for the protection of subjects.

The ACTIVE/ADIPOSE study enrolled prevalent hemodialysis patients from 14 centers in San Francisco CA and Atlanta GA from June 2009 through August 2011^25^. Blood was drawn prior to dialysis every six months, separated by centrifugation at the local facility and then shipped on dry ice to the core laboratory at the University of California Davis where it was thawed once, aliquoted and then frozen over liquid N_2_ until assay. Body composition was measured as Body Mass Index, waist circumference. Stored samples that had not been previously thawed were obtained from patients enrolled in the ACTIVE/ADIPOSE study^26^ with type 2 diabetes mellitus receiving hemodialysis (HD) who were hospitalized for an infectious event within 4 to 56 days (mean 24.5 ± 23.6 days) of the blood collection (n = 13), and patients with diabetes mellitus receiving HD who remained infection-free for at least the subsequent two years (median 799.5, 25^th^ percentile 800 75^th^ percentile 840) (n = 13). The samples were grouped for analysis based on the time until first infectious event. Two samples (one from the HD+ group and one from the HD- group) did not have adequate HDL levels to complete the immune cell assays at the 30 mg/dL HDL cholesterol final concentration and were therefore excluded from analysis.

### HDL Isolation by Ultracentrifugation

Preparation of HDL was performed as described previously^21^ and scaled up for preparative purposes. Potassium Bromide (KBr) densities of 1.019, 1.063 and 1.340 g/mL were freshly made weekly and verified using the Densito30PX portable densitometer (Mettler Toledo, Columbus, OH, USA). Briefly, plasma samples (1.9 mL) were previously adjusted to d = 1.019 by adding a concentrated KBr solution (d = 1.340) and underlaid to KBr solution of d = 1.019 for a final volume of 4.7 mL tube (OptiSeal, Beckman Coulter). Ultracentrifugation was performed using a Beckman Optima MAX-TL equipped with a TLA-110 fixed-angle rotor (Beckman Coulter) for 2 h, at 657,000× g and 15 °C. The VLDL-IDL fraction of density lower than 1.019 was recovered from the top of the tube (1.9 mL), and the remaining infranate was adjusted to d = 1.063 and underlaid with KBr solution of the same density (d = 1.063) followed by ultracentrifugation for 3 h. After centrifugation, the LDL supernate fraction (1.9 mL) was collected and the infranate containing the HDL fraction adjusted to d = 1.210, and 4.7 mL subjected to ultracentrifugation for 3.5 h. The HDL fraction (1.21–1.063 g/mL) was collected from the top of the tube (1.0 mL) and subjected to diafiltration using Amicon ultra-3K centrifugal filter devices. During this process the HDL fractions were desalted by washing out the KBr salt with water (Optima LC/MS) by two consecutive steps of centrifugation for 25 min at 4 °C and 14,000× g. The concentrated HDL fraction was recovered in Optima Water for glycoproteomic analysis and in PBS for the immune experiments.

### HDL Fraction Analyses

Each of the following was measured in the separated HDL fractions. HDL cholesterol was measured using a colorimetric Total Cholesterol assay from Cellbiolabs (Cellbiolabs, San Diego CA). ApoA-I was measured using the Cellbiolabs human ApoA-I Elisa kit (Cellbiolabs, San Diego CA). Total Endotoxins were measured using a chromogenic LAL (*Limulus* Amebocyte Lysate) assay (Pierce Biotechnologies, Grand Island, NY) in a subset of 18 samples to verify that there was no significant contamination of the isolated HDL with LPS, and that there were no differences in the amount of LPS in the isolated HDL fractions between groups. There were negligible levels of endotoxin in the samples and there were no differences in endotoxin levels between groups (data not shown). LBP was measured using an electrochemiluminescent assay (Meso Scale Discovery, Rockville, MD). ApoA-I and LBP were normalized to total cholesterol in the HDL fraction.

### Glycoproteomic Analysis

Apolipoprotein C-III (Apo C-III), α-1-antitrypsin (A1AT), α-2-human serum glycoprotein (A2HSG) from human plasma and recombinant human apo serum amyloid A (SAA) were purchased from Sigma-Aldrich (St. Lous, MO). Sequencing grade modified trypsin and dithiothreitol (DTT) were purchased from Promega (Madison, WI). Iodoacetamide (IAA) was purchased from Sigma-Aldrich (St. Louis, MO). All the trypsin digestion experiments were carried out in freshly made 50 mM NH_4_HCO_3_ solutions. For the tandem mass spectrometry experiments, 20 ug of ApoC-III, A1AT, A2HSG, and SAA in 100 uL ammonium bicarbonate solutions were reduced using 2 uL of 550 mM DTT in 60 °C for 50 min then alkylated using 4 uL of 450 mM IAA in the dark at room temperature for 30 min and digested at 37 °C overnight using 1ug of trypsin. Digestion reactions were stopped by placing the samples in −20 °C for 1hr. the resulting tryptic digests were injected into the LC QE -Orbitrap instrument without any sample clean up. 10 uL of HDL samples were added to 90 uL of the buffer solution and digested using the above procedure but 2 ug of the trypsin was used for the digestion. Byonic software (San Carlos, CA) was used to identify the peptides and the glycopeptides using accurate mass and fragment ion patterns.

For the peptide and glycopeptide quantitation, the four protein standards were pooled together and digested using trypsin. Serial dilution was performed and 6 concentration levels with ratios 1:2:2.5:2:5:2 were created for the protein concentration measurements, the highest concentration being 0.1810. The peptides and glycopeptides were analyzed using Agilent 1290 infinity LC system coupled to an Agilent 6490 triple quadrupole (QqQ) mass spectrometer (Agilent technologies, Santa Clara, CA). The analytical column used for UPLC separation was an Agilent eclipse plus C18 (RRHD 1.8 μm, 2.1 × 100 mm) connected to an Agilent eclipse plus C18 pre-column (RRHD 1.8 μm, 2.1 × 5 mm). 2 uL of the HDL sample solutions were injected and analyzed using a 14-minute binary gradient as follows: 0.0−0.5 min, 2% B; 0.5−5 min, 2−15% B; 5−10 min, 15−44% B; 10−12.1 min, 44−100% flush at 100% B for 1.1 min and equilibrium for 0.8 min at 2% B. Solvent A was composed of 3% acetonitrile, 0.1% formic acid in while solvent B was 90% acetonitrile, 0.1% formic acid all in nano pure water (v/v). The sample flow rate was 0.5 mL/min.

The MS parameters for the glycopeptides have been optimized before in our lab^27^. Briefly, positive ion mode was used to ionize the samples at unit resolution. The dynamic multiple reaction monitoring (MRM) mode that monitors analyte transitions only when they are eluting from the LC was used for quantitation. The collision energies for each peptide and the glycopeptide transitions were optimized to achieve optimum sensitivity. Unique peptides for each protein and the common glycan oxonium fragments, such as m/z 204.08 (HexNAc), 366.14 (Hex1HexNAc1), 292.09 (Neu5Ac) and 274.09 (Neu5Ac –H_2_O) were used to quantify proteins and glycopeptides respectively. Protein concentrations (in ion counts) were normalized to the total cholesterol in the HDL extract. Each glycopeptide concentration (in ion counts) was normalized to the total amount of its parent protein (in ion counts). Agilent Mass Hunter quantitative analysis software was used to analyze the MRM data with limit of detection signal to noise ratio (S/N) ≥ 3 and limit of quantitation S/N ≥ 6. Glycan composition was designated by the number of hexose (Hex) residues, N-acetylhexosamine (HexNAc) residues, fucose (Fuc) residues, and N-acetylneuraminic acid (NeuAc) residues. As an example, a glycan containing 5 Hex, 4 HexNAc, 0 Fuc and 2 NeuAc residues was designated by the name 5–4–0–2. Nomenclature for each glycopeptide includes the protein name, followed by the site of the glycan attachment, and the glycan composition (e.g. A1AT_70_5402).

### Monocyte Assay

Heparinized blood samples from healthy adult donors were purchased from BloodSource (Mather, CA). Peripheral blood mononuclear cells (PBMCs) were isolated using Ficoll-Paque density gradients (GE Healthcare Bioscience). Cells were resuspended in RPMI 1640 tissue culture medium supplemented with 5% fetal bovine serum and 100 U/ml penicillin-streptomycin (all from Gibco BRL, Gaithersburg, Md.), washed in the medium and then were counted using an automated TC20 cell counter (Bio-rad). Monocytes were positively isolated from PBMCs using BD IMag^TM^ CD14 magnetic particles (BD Biosciences) according to the manufacturer’s protocol. Monocytes were cultured in duplicate in a 96 well plate (1~2 × 10^6^ cells/ml, 200 ml/well) with or without 10 ng/ml LPS (LPS-EK from *E. coli* K12, InvivoGen) in the presence or absence of HDL from individual subjects for 24 hours, with a 0 LPS, 0 HDL negative control. Cholesterol concentrations in the separated HDL fractions were measured as described above, and used to concentrate each sample to a final concentration of 30 mg/dL cholesterol in the well. Culture supernatant aliquots for cytokine measurement were stored at −80 °C until analysis.

### Cytokine Measurement

We selected IL-6 as a prototypical cytokine to measure as it is one of the classic cytokines released by monocytes in response to LPS and its impact on plaque development and morphology has been well described^28^,^29^. Culture supernatant samples were diluted (1:2) using culture medium. Assays were performed according to the manufacturer’s instructions. Briefly, 50 μl of each diluted sample were added to a suspension of beads coated with primary antibody against IL-6 in each well of an assay plate and incubated for 30 min at room temperature in the dark with shaking at 450 rpm. After the incubation, the beads were washed 3 times and subsequently reacted with a mixture of biotin-conjugated secondary antibody. After a 30 min reaction, the beads were again washed and re-suspended in assay buffer containing streptavidin-phycoerythrin (Str-PE). After 10 min of agitation at room temperature in the dark, the beads were washed and re-suspended in assay buffer. Concentrations of IL-6 were measured using a Bio-Plex 200 (Bio-Rad Laboratories, Inc., Hercules, CA, USA) with known concentrations of human IL-6 standard (Bio-Rad). Bio-Plex Manager software (Bio-Rad) was used to calculate the concentrations of IL-6 in each well.

### HDL Particle Size Measurement

HDL size was measured using the 90Plus/BI-MAS Particle Size Analyzer (Brookhaven Instruments Corp., Holtsville, NY) and the MAS OPTION Software, which combines the photon correlation spectroscopy (PCS) technique with Quasi-elastically scattered light (QELS). Twenty five ul aliquots of native HDL extracts were diluted to make up to a 1 mL aliquot of PBS in a Sarstedt Acryl Cuvette (Sarstedt AG & Co, Germany), and placed in the cuvette holder in the Particle Size Analyzer. Three-minute measurements were collected, and processed by the MAS OPTION software forming an autocorrelation function returning size in nm.

#### Statistical Analyses

All statistical analyses were performed using R statistical software (R core development, Vienna) and JMP (R) Pro11.2.0 64-bit (SAS institute, Cary NC), and Microsoft Office Excel (Microsoft, Seattle WA). Shapiro-Wilk tests were used to determine the normality of data distribution. When data were normally distributed, ANOVA with Tukey’s post hoc was used. When data were not normally distributed, non-parametric van der Weardan’s test, followed by Steel-Dwaas post hoc tests were used. The quantity of IL-6 in each well where HDL was added was converted to a % change in IL-6 relative to the 0 HDL treatment to calculate IL-6 response using the following formula:


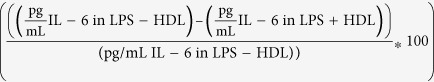


The negative control wells (i.e. 0 HDL and 0 LPS) had no cytokine response. The % change values were used to categorize individual subjects’ HDL into either enhancement (where IL-6 response was a positive value) or suppression (where IL-6 response was a negative value) of IL-6 response. Van der Wearden’s non-parametric tests were used to identify group differences between control, MetS and HD subjects, as well as IL-6 response groups. Steel-Dwaas multiple comparison tests were used with a p < 0.05 as an indicator of significance.

Partial Least Squares Discriminant Analysis (PLS-DA) was used to explain the variance between clinical groups (control vs MetS vs HD) as well as IL-6 response groups (IL-6 increased vs. decreased). A random 2/3^rd^ subset was chosen to be the training set and the other 1/3^rd^ to be the test set for cross-validating each PLS model. External cross-validation was done by extracting PLS models from three repeated runs of the test-training set validation. The models were built using the NIPALS method, which extracts one factor at a time and was used to evaluate which variables primarily explain the variation between groups. The optimal models were chosen based on the goodness of prediction statistic *Q2*. In addition, the coefficients of multiple determination *R*^*2*^for independent and dependent variables were also used to determine the goodness of fit. Both these statistics were also considered while evaluating variable selection. Variables that had a Variable Importance Plot (VIP) score of >1 were considered as the primary variables driving the separation between groups. The model diagnostics (Q2, R^2^X, R^2^Y) are all reported as mean ± standard deviation.

Glycopeptides for each protein were also summed based on whether they were sialylated, fucosylated, or undecorated, and then mol percentages were calculated for these categories including mol% non-fucosylated, mol% monofucosylated, mol% difucosylated, mol% non-sialylated, mol% mono-sialylated, mol% di-sialylated, mol% undecorated (i.e. neither fucosylated nor sialylated), mol% fucosylated + sialylated (i.e. fucosylated and sialylated). In each case, the mol% category was calculated as the amount of the category divided by the total glycopeptides for each protein (e.g. A1AT mol% monofucosylated was calculated as the total of monofucosylated glycopeptides on A1AT divided by the total glycopeptides on A1AT). The individual glycopeptide data and mol% categories were also evaluated to determine differences between the IL-6 response groups, and between the HD patients who developed infections and those who did not, also using the van der Wearden test, with Steel-Dwaas correction for pair-wise comparison errors.

## Results

### Anthropometric and Clinical Characteristics

Table 1 compares controls to the MetS and HD groups. The control group had a significantly lower BMI compared to the MetS group (p < 0.001), as well as the HD group (p < 0.001). The control group was also significantly younger than the HD (p < 0.001) group. No differences were found between groups in HDL particle size. Cholesterol in the isolated HDL was significantly higher in the control compared to the HD group (p = 0.031). ApoC-III and LBP were higher in both HD and MetS compared to controls (p < 0.01 for both). SAA was higher in the HD group compared to control and MetS groups (p < 0.001 for both). The MetS group had higher circulating triglycerides compared to HD (p < 0.01). Plasma TG and CRP levels were not measured in the control subjects. The MetS subjects had lower systolic blood pressure compared to HD (p < 0.01), as did the controls (p < 0.01).

### Glycoprotein and Site-Specific Glycopeptide Composition in the Clinical Groups

**Figure 1:**
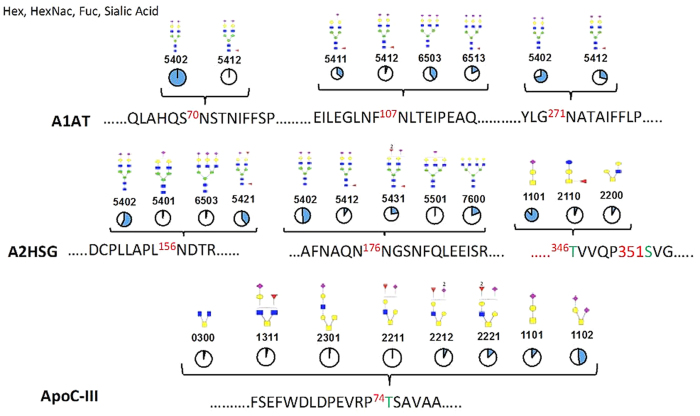
Protein backbone and putative structures for N and O-glycopeptides including their relative abundances within the site. There were three N-linked glycosylated sites in A1AT, 2 N-linked and 2 O-linked glycosylation sites in A2HSG while ApoC-III had only one site populated with O-linked glycans. The modified amino acid number is shown in red and the residue modified with O-glycans is colored green. The glycan monosaccharides annotation includes (blue ◼) N-acetylglucosamine (HexNAc), (green ●) mannose, (yellow ●) galactose, (○) hexose, (red ▲) fucose, (purple ◆) N-acetyl neuraminic acid. The four number glycan code represents the number of hexoses, HexNAc, fucose, and N-acetyl neuraminic acid residues in that order. The pie charts shows the relative abundances of different glycans within a given protein standard site.

The glycopeptides monitored on each of the 3 proteins (i.e. A1AT, A2HSG, and ApoC-III), as well as the relative distribution of glycans on each site as measured in the protein standards are depicted in Fig. 1. As shown in the figure, there were three N-linked glycosylated sites in A1AT, 2 N-linked and 2 O-linked glycosylation sites in A2HSG, and one O-linked glycosylated site in ApoC-III. For each glycan composition a putative structure is depicted, with the pie charts showing the relative abundances of different glycans within a given site based on analyses of the protein standards.

**Figure 2:**
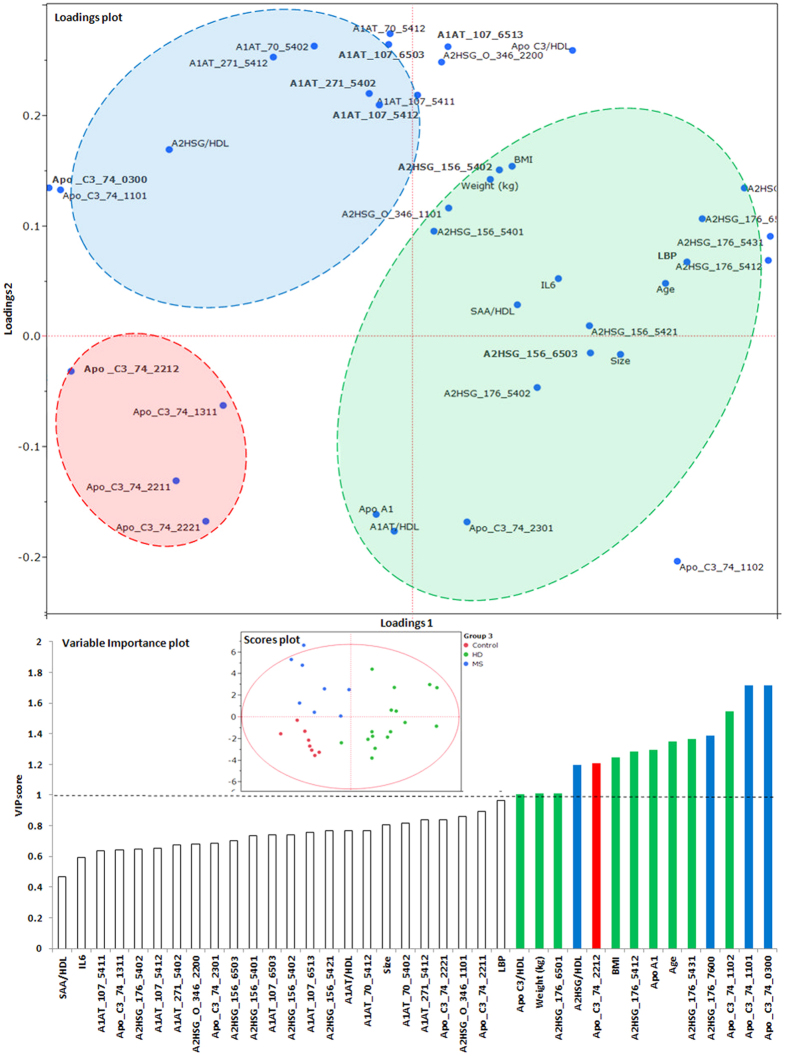
PLS-DA model Loadings, Scores and Variable importance plots discriminating anthropometric, clinical and glycoproteomic variables in n = 50 individuals based on clinical groups – controls, diabetic patients on hemodialysis (HD), and subjects with metabolic syndrome (MetS). The loadings plot displays the anthropometric, clinical and glycopeptide variables that explain the variance between the groups (highlighted using blue for MetS, red for controls, and green for HD), while the scores plot indicates the groups of participants in each of the clinical group, and their distribution in this dimensional space. The X and Y axes represent the X-loadings 1^st^ and 2^nd^ components respectively. The variable importance plot identified 14 primary variables (highlighted using colors blue, green and red) that drive the difference between the clinical groups. The model had a Q2 of 0.92, explaining 62% of variance in X variables, and 79% of variance in Y variables.

Figure 2 displays the variance in anthropometric, clinical and glycopeptide variables between the clinical groups. In the loadings plot, the blue highlighted section corresponds to variables associated with the blue dots in the scores plot, belonging to the MetS group. Similarly, the green highlight and green dots represent the HD group, and the red highlight and red dots represent the control group. The variable importance plot also has variables colored blue, green or red, with variables that have a VIP > 1 deemed significant in the discriminant analysis corresponding to the Mets, HD and control groups respectively. The HD group was defined by higher ApoC-III, ApoA-I, body weight, BMI, age, as well as the glycopeptides A2HSG_176_6501, A2HSG_176_5412, A2HSG_176_5431, and Apo C3_74_1102. MetS was characterized by higher A2HSG, A2HSG_176_7600, ApoC3_74_1101, and ApoC3_74_0300. Controls were marked by higher ApoC3_74_2212. The PLS-DA model had a Q2 value of 0.92+/− 0.01, an R^2^X value of 0.62+/− 0.01 and R^2^Y value of 0.79+/− 0.01. The number of variables with VIP values >1 was 14, and given the high Q2 and R^2^X and R^2^Y values, these VIP variables are strong predictors of separation into the MetS, HD and control groups.

**Figure 3:**
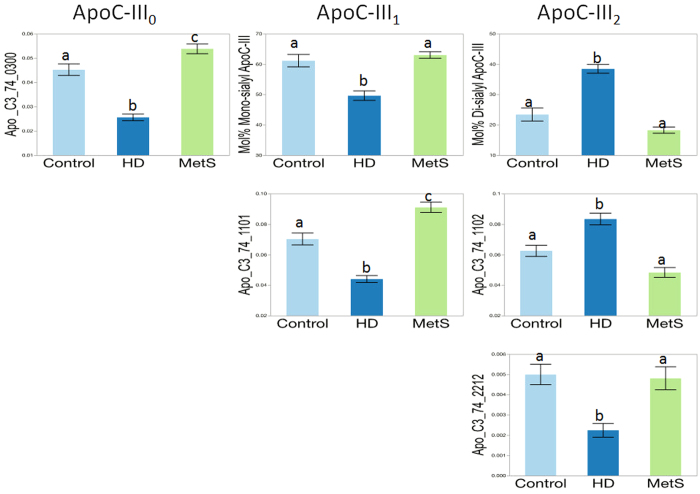
Apolipoprotein C-III (ApoC-III) glycosylation patterns in HDL isolated from control, diabetic patients on hemodialysis (HD), and metabolic syndrome (MetS) subjects. Significant differences (‘a’, ‘b’ and ‘c’ subscripts indicate individual group differences at p < 0.05). ApoC-III_0_, non-sialylated; ApoC-III_1_, mono-sialylated; ApoC-III_2_, di-sialylated.

Non-parametric univariate analysis also showed significant differences between the clinical groups (control vs MetS vs HD) in the glycosylation patterns for the three glycoproteins that were characterized. The key results for ApoC-III are shown in Fig. 3. The non-sialylated isoform of ApoC-III (ApoC-III_0_) was represented by the single undecorated glycan 0300 on site 74, and was significantly lower in HD compared to controls and MetS subjects (p < 0.001 for both) and higher in MetS than controls (p = 0.031). The mol% of mono-sialylated ApoC-III isoforms (ApoC-III_1_) was significantly lower in HD than control and MetS subjects (p ≤ 0.01 for both). The mono-sialylated glycan 1101 on site 74 was lowest in HD, highest in MetS, and intermediate in control subjects (p ≤ 0.005 for three). The mol% of di-sialylated ApoC-III isoforms (ApoC-III_2_) was significantly higher in HD than controls and MetS subjects (p ≤ 0.001 for both), with levels being lowest in MetS (though the difference between Mets and controls was NS). The di-sialylated glycan 1102 on site 74 was also increased in HD compared to controls and MetS subjects (p ≤ 0.01 for both) with levels being lowest in MetS (although again, the difference between MetS and controls was NS). However the opposite pattern was seen with the di-sialylated glycan 2212, which was lower in HD than in controls and MetS subjects (p ≤ 0.005 for both). Thus, while MetS subjects had the lowest levels of sialylation in ApoC-III, HD patients had a specific enrichment in ApoC-III_2_, with controls being intermediate.

**Figure 4:**
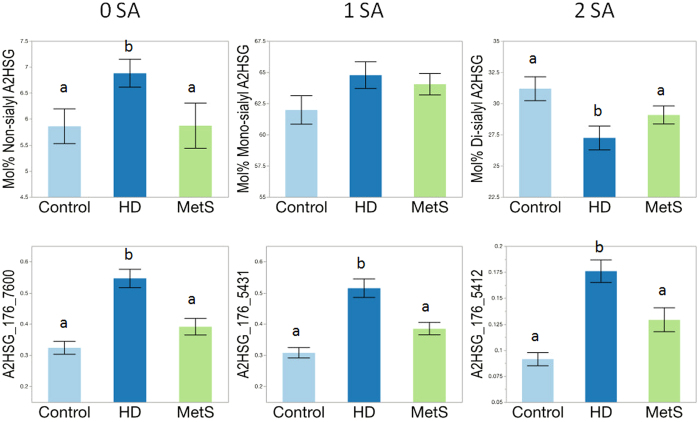
Glycosylation patterns of α-2HS-glycoprotein (A2HSG, fetuin A) in HDL isolated from control, hemodialysis (HD), and metabolic syndrome (MetS) subjects. 0, 1 and 2 SA represent non-, mono- and di-sialylated glycoforms. Significant differences (‘a’ and ‘b’ subscripts indicate individual group differences at p < 0.05).

Results for A2HSG are shown in Fig. 4. The mol% of non-sialylated A2HSG was significantly higher in the HD subjects compared to controls (p = 0.035). The mol% mono-sialylated A2HSG glycoforms did not reach statistical significance but showed a trend toward an increase in HD compared with control subjects, with MetS subjects being intermediate. On the other hand, the mol% of di-sialylated A2HSG was significantly lower in HD compared to controls and MetS (p < 0.05). Both the individual non-sialylated glycan 7600 on site 176 and the di-sialylated glycan 5412 on site 176 were significantly increased in HD compared with control and MetS subjects (p < 0.005 for non-sialylated and p < 0.01 for di-sialylated for all three), as was the mono-sialylated glycan 5431 on site 176 (p < 0.01 for both). Thus, HD patients generally had less sialylation in A2HSG but increased levels of the specific glycan 5412.

HD patients had less non-fucosylated, more monofucosylated, and less di-sialylated A1AT compared to MetS and controls (p < 0.05), with no differences between MetS and controls (Supplemental Figure S1). These data indicate that an enrichment in fucosylated glycans on A1AT may be characteristic of HD patients.

### IL-6 Response Groups

**Figure 5:**
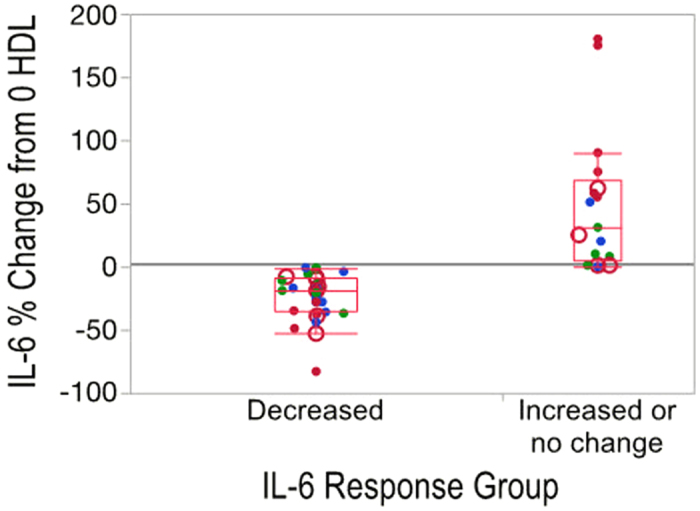
IL-6% response in the decreased vs. increased or no change groups. Experimental subjects with different health conditions were grouped based on their HDL pre-exposure to standard monocytes causing decrease vs increase or no change in IL-6 secretion upon lipopolysaccharide (LPS) stimulation. Control subjects (green circles); metabolic syndrome subjects (blue circles); diabetic patients receiving hemodialysis who had an infectious hospitalization event (filled red circles) and those who did not have an infectious event (open red circles).

**Figure 6:**
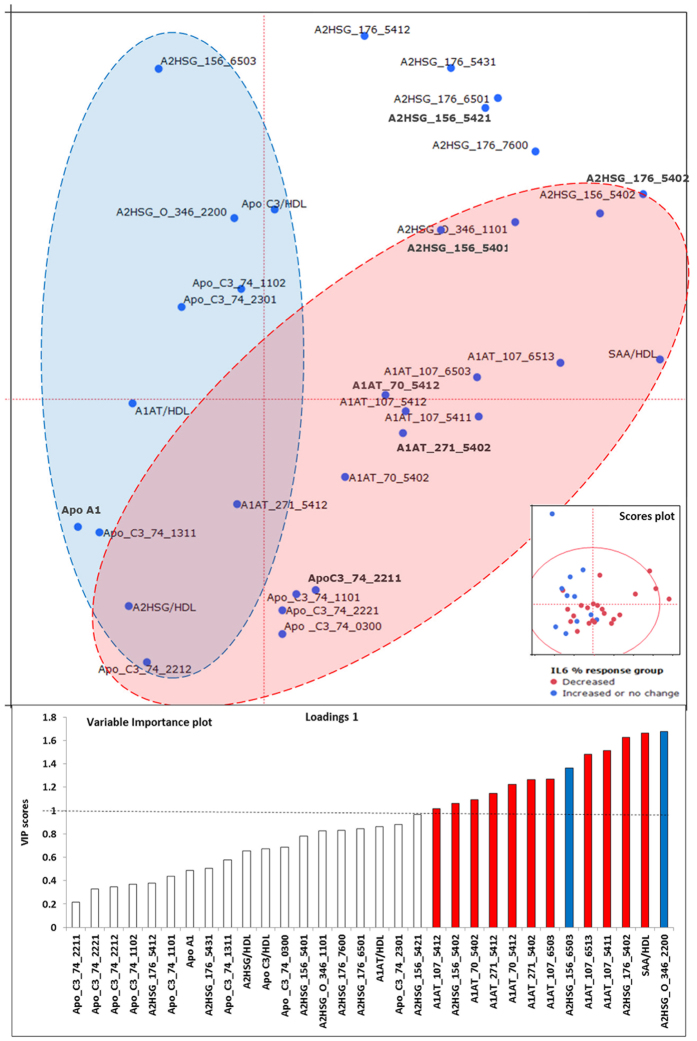
PLS-DA model Loadings, Scores and Variable importance plots discriminating glycoproteomic variables in n = 50 individuals based on IL-6 response groups (percentage change in IL-6 secretion with HDL pre-incubation in LPS-stimulated monocytes) – Decreased (color red) or Increased or no change (color blue). The loadings plot displays the glycopeptide variables that explain the variance between the groups, while the scores plot indicates the groups of participants in each of the IL-6 response group, and their distribution in this dimensional space. The variable importance plot identified 13 primary variables (highlighted using colors blue and red) that drive the difference between the IL-6 response groups. The model had a Q2 of 0.14, explaining 37% of variance in X variables, and 31% of variance in Y variables.

Out of the 50 HDL samples analyzed 33 decreased and 17 increased or did not change IL-6 secretion in monocytes in response to LPS stimulation (Fig. 5). The grand mean in IL-6 response across all 50 HDL samples was no effect on IL-6 secretion. However, the range in responses across the 50 individual HDL samples was from an 83% decrease to a 180% increase in IL-6 relative to 0 HDL. Given this wide range of IL-6 responses to HDL we separated responders versus non-responders to evaluate how the HDL glycoforms differed between these groups. Thus, subjects were divided into those whose HDL increased (i.e. IL-6% change ≥0) vs. decreased (i.e. IL-6% change <0) IL-6 response. The mean response of the group in which HDL increased IL-6 secretion was a 50% increase in IL-6, and the mean response in the group that decreased IL-6 secretion was a 23% decrease in IL-6. There was a mixture of samples from each of the 4 clinical groups (controls, MetS, HD+ and HD- subjects) in both of the IL-6 response groups, suggesting that the ability of HDL to suppress IL-6 secretion in LPS-stimulated monocytes may not be directly linked to the clinical characteristics of the patient but rather, more likely, the composition of their HDL particles. In support of this hypothesis, a multivariate model for IL-6 groups could not be fitted by the NIPALS PLSDA algorithm, using a 1/3^rd^ 2/3^rd^ training and test set paradigm, when the anthropometric, clinical, and glycopeptide data were included. However, the PLS DA models using only glycopeptides to predict the IL-6 response groups were successful (Fig. 6). The red and blue highlighted regions in the loadings plot correspond to the red and blue dots in the scores plot denoting decreased (red) and increased or no change in IL-6 (blue) groups. Similar to Fig. 2, the VIP variables are highlighted using red and blue colors to indicate decreased and increased IL-6 response groups. The Q2 value was 0.14 +/− 0.01, and R^2^ values were 0.38 +/− 0.17 and 0.31 +/− 0.21 for the X and Y variables respectively, with 13 variables having VIP values > 1.

**Figure 7:**
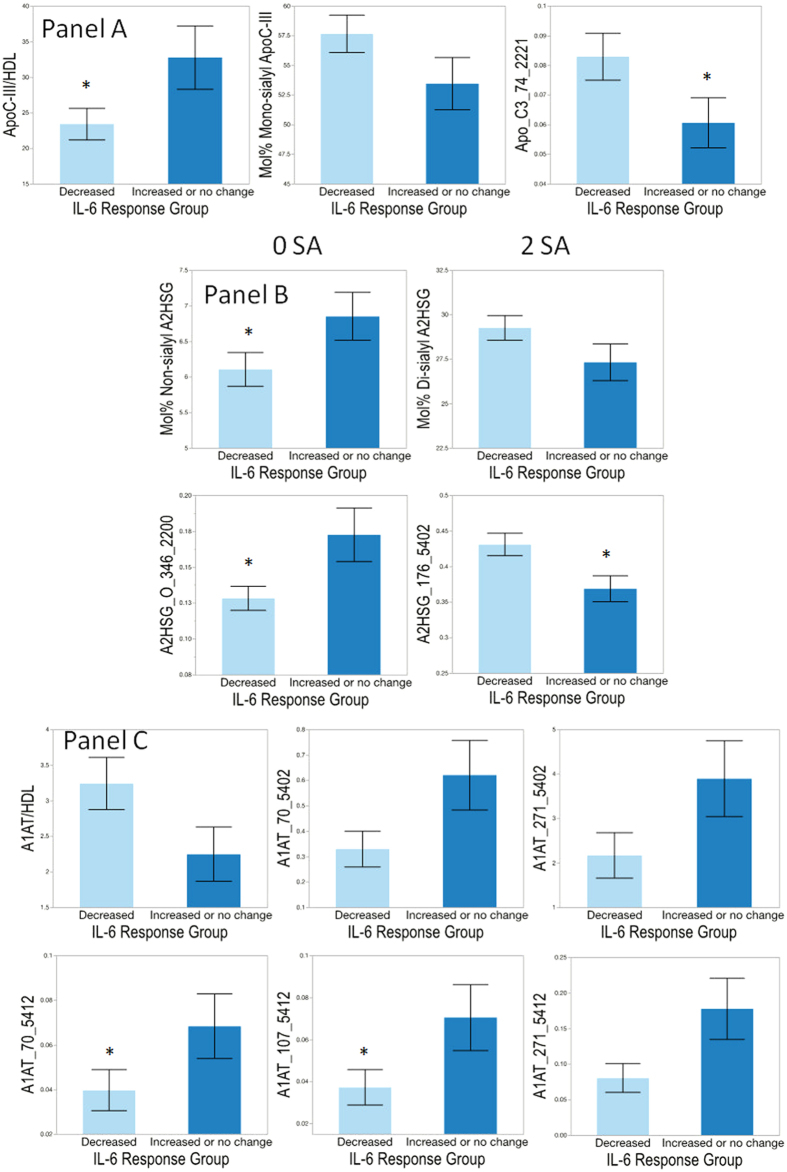
Panel A: Differences in protein amounts and glycosylation patterns of apolipoprotein C-III (ApoC-III) by interleukin 6 (IL-6) response group comparing HDL that decreased vs. increased or did not change secretion of IL-6 in LPS-stimulated monocytes. Panel B: Differences in glycosylation patterns in α-2HS-glycoprotein (A2HSG, fetuin A) between IL-6 response groups comparing isolated HDL that decreased vs. increased or led to no change in IL-6 secretion in LPS-stimulated monocytes. Panel C: Differences in protein amounts and glycosylation patterns of α-1-antitrypsin (A1AT) by IL-6 response group comparing HDL that decreased vs. increased or did not change secretion of IL-6 in LPS-stimulated monocytes. Differences are indicated using ‘*’ at p < 0.05.

There were no differences in most of the anthropometric and clinical parameters (blood pressure, BMI, weight, etc.) and several of the measured proteins (LBP, ApoA-I, SAA, A2HSG) between the IL-6 response groups (data not shown). ApoC-III was higher in HDL that increased IL-6 secretion (p = 0.043), while the level of the individual mono-sialylated glycan 2221 was lower in HDL that increased IL-6 secretion (p < 0.05) (Fig. 7, Panel A). Mol% monosialylated ApoC-III_1_ also showed a trend toward being lower in the HDL that increased IL-6 secretion. HDL that increased IL-6 secretion in stimulated monocytes had higher levels of non-sialylated A2HSG isoforms though these did not reach statistical significance (Fig. 7, Panel B). Illustrating this pattern, the individual non-sialylated O-glycan 2200 on site 346 (p = 0.017) was lower and the di-sialylated N-glycan 5402 on site 176 (p = 0.015) was higher in those HDL that increased IL-6 response. A1AT was lower (although it did not reach statistical significance) in HDL that increased IL-6 response (Fig. 7 Panel C), and there were significantly higher levels of two specific disalylated glycans. These two glycans were the mono-fucosylated glycoform 5412, which was higher on two different sites (70 and 107, p < 0.05 for both), in HDL that increased IL-6 response. Thus, HDL that increased IL-6 secretion were enriched in the glycan 5412 on A1AT, had lower levels of sialylation in A2HSG as well as lower levels of the glycan 5402 on A2HSG, and increased levels of total ApoC-III and decreased ApoC-III_1_.

IL-6 response was positively correlated with plasma CRP (*p* = 0.03, r^2^ = 0.133) (Supplemental Figure S2; correlation calculated with CRP data from the 37 HD and MetS subjects), indicating that the higher the overall inflammation status the more pro-stimulatory the HDL that are associated with that plasma sample.

### Differences Between HD Patients With vs. Without Infectious Hospitalization Event

**Figure 8:**
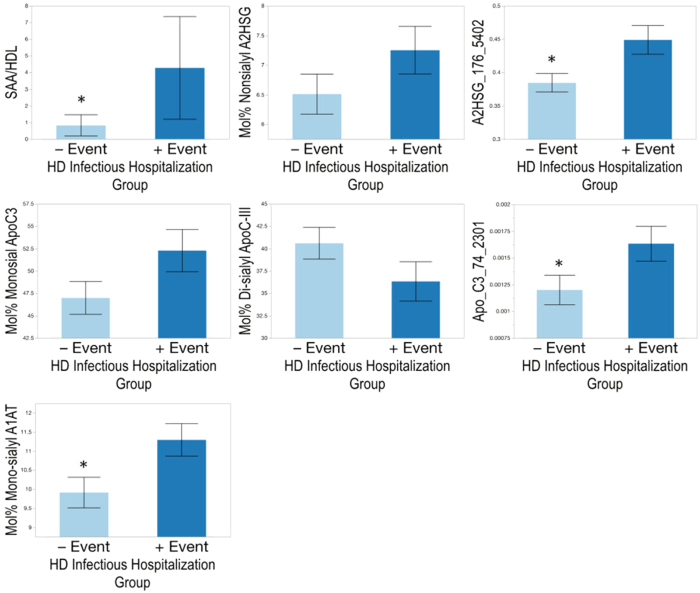
Differences in protein amounts of serum amyloid A (SAA) and glycosylation patterns of α-2HS-glycoprotein (A2HSG, fetuin A), apolipoprotein C-III (ApoC-III), and α1-antitrypsin (A1AT) by hemodialysis (HD) patient infectious hospitalization group comparing HDL from subjects who had an infectious hospitalization event within 60 days vs. those who had no event. Differences are indicated using ‘*’ at p < 0.05.

We also conducted subgroup analysis on the HD group separating HD patients who went on to be hospitalized for an infectious event within 60 days of their baseline visit (HD+) to those who had no events during almost three years of follow-up (HD−). There were no significant differences between the HD(−) and HD(+) groups in the anthropometric or clinical characteristics (Table 2). The total amount of SAA was significantly higher in HD+ subject HDL compared to HD-(p < 0.05) (Fig. 8) but only when 3 outliers were removed from the analysis. HD+ patients also had higher levels of the individual di-sialylated glycan 5402 on site 176 of A2HSG (p < 0.05). HDL from HD+ subjects also had higher levels of mono-sialylated ApoC-III but lower levels of di-sialylated ApoC-III, though this did not reach statistical significance while the individual glycan 2301 on site 74 was increased in HD+ (p = 0.038). The HD+ subjects also had higher levels of mono-sialylated A1AT (p = 0.023) and lower levels of trisialylated A1AT though this did not reach statistical significance. Thus, HD+ patients had lower levels of sialylation across the three glycoproteins studied but higher levels of the glycan 5402 at site 176 of A2HSG.

## Discussion

Our results indicate that the glycoprotein composition of important HDL-associated proteins does indeed successfully differentiate between clinical groups across the range of insulin sensitivity from normal, to pre-diabetic, to diabetic with renal failure, especially Apo C-III isoforms. HDL from HD patients were enriched in SAA, LBP, ApoC-III, di-sialylated ApoC-III (ApoC-III_2_) and desialylated A2HSG. Based on the prototypical assay of HDL’s immunomodulatory capacity (to modulate IL-6 secretion in LPS-stimulated monocytes), HDL that increased IL-6 secretion were enriched in ApoC-III, di-sialylated glycans on A1AT and desialylated A2HSG, and depleted in mono-sialylated ApoC-III (ApoC-III_1_). Subgroup analysis on HD patients who experienced an infectious hospitalization event within 60 days vs. those with no event showed that HDL from HD+ patients were enriched in SAA but had lower levels of sialylation across glycoproteins.

ApoC-III glycoforms were key in differentiating between the clinical groups. ApoC-III was higher in HDL of both HD and MetS patients compared to controls, confirming previous reports of increased levels of ApoC-III in HDL of diabetics^30^ and patients with renal failure^31^,^32^. ApoC-III is known to exist in three different isoforms corresponding to differential migration on a gel due to differences in the number of sialic acid residues. ApoC-III containing no sialic acid is denoted as ApoC-III_0_, 1 sialic acid as ApoC-III_1_, and 2 sialic acid residues as ApoC-III_2_^33^. Our data indicate an enrichment in ApoC-III_2_ and loss of ApoC-III_0_ and ApoC-III_1_ isoforms in HD patients. ApoC-III_2_ isoforms have been shown to associate with LDL particles, in particular small dense LDL particles^33^, and dyslipidemia in HD patients is associated with an enrichment in small dense LDL^34^. Thus, it is possible that the isolated HDL fractions from the HD subjects were contaminated with small dense LDL. Alternately it is possible that there is an increased exchange of apoproteins between LDL and HDL in the blood of HD patients, or that there is transcriptional regulation or an effect of treatment on ApoC-III glycosylation that leads to this phenotype in HD patients. MetS subjects, in contrast, had significantly higher levels of ApoC-III_0_ and ApoC-III_1_ isoforms, and a trend toward lower levels of ApoC-III_2_ isoforms relative to controls, suggesting a loss of sialylation in ApoC-III compared with controls, similar to previously published observations in MetS patients^23^. Our data highlight the importance of characterizing the glycosylation profile, with glycan specificity. For example, in spite of the fact that HDL-associated ApoC-III from HD patients were enriched in ApoC-III_2_ glycoforms in general, the di-sialylated glycan 2212 was depleted, and may be a specific marker of aberrant glycosylation associated with HD.

We observed that HD patients had the lowest levels of di-sialylated and highest levels of mono- and non-sialylated A2HSG glycans, with MetS subjects intermediate between HD and controls. Thus, in general, loss of sialylation of A2HSG was associated with progressive metabolic and kidney dysfunction. Saroha A *et al*. observed a similar trend of desialylation to be associated with rheumatoid arthritis^35^. Yet, HD patients had an increased level of the di-sialylated glycan 5412 on A2HSG, which may be a marker of pathway-specific alterations in glycosylation.

Our data also indicate that HDL that induced a more pro-stimulatory phenotype in monocytes were enriched in ApoC-III but that this ApoC-III was depleted of mono-sialylated glycans, which are the predominant glycoform in normal healthy subjects. Similarly, we observed a loss of sialylation on A2HSG in HDL that induced a pro-stimulatory phenotype in monocytes. In contrast, HDL that induced a pro-stimulatory phenotype were enriched in two di-sialylated glycans (5402 and 5412) across multiple N-glycosilation sites of A1AT. However, since we did not find a significant difference in the mol% of di-sialylated glycans in A1AT, it is possible that rather than a general trend toward higher sialylation of A1AT, there is a specific change in the content of these two specific di-sialylated glycans on A1AT that affects the functionality of HDL. A1AT is known as an acute phase reactant that has multiple functions including its well-known antiprotease function, as well as newer functions in modulating immune response, generally in the direction of suppressing immune activation and reducing inflammatory signaling (reviewed in ref. 36).

Our data suggest that HDL glycoproteomic profiling has the potential to lead to discoveries into mechanisms regulating the immunomodulatory function of HDL. More studies are needed, especially ones powered to account for gender and age in addition to clinical characteristics, to determine the aspects of HDL glycoprotein composition that specifically affect HDL’s immunomodulatory function. In addition, in this study we used a prototypical assay of HDL’s immunonodulatory capacity using an LPS-stimulated monocyte model, yet it is known that HDL interact with a broad array of immune cell types, with a wide range of specific immunomodulatory effects (reviewed in ref. 37). A larger repertoire of HDL immunomodulatory functional assays is needed to more comprehensively characterize the effects of changes in HDL composition on its capacity to modulate the immune system.

In our study we found increased levels of SAA in HDL from HD patients who had an infectious hospitalization event within 60 days. Previous studies have found the presence of SAA on HDL to be associated with loss of anti-inflammatory capacity^14^,^15^, and increased binding to vascular proteoglycans^38^. Our results also indicate that HDL isolated from HD patients who went on to have an infectious hospitalization event were generally depleted of sialic acid across all three glycoproteins characterized. These data support the hypothesis that loss of sialic acid might be associated with loss of protection against infection.

The effects of sialylation on protein function have been documented. For example, it has been demonstrated that immunoglobulin (Ig) glycosylation is linked with the binding affinity of the Ig to its receptors on immune cells (reviewed in ref. 20). Fucosylated, sialylated N-glycans on IgG are associated with anti-inflammatory properties compared to undecorated, ungalactosylated N-glycans, because these modifications modulate the binding affinity of the IgG to activating vs. inhibiting Fc receptors^20^. A2HSG (also known as fetuin A) and A1AT were found to be differentially glycosylated in chronic pancreatitis and pancreatic cancer^39^. However, to our knowledge, our study is the first to examine the effects of glycosylation, particularly site-specific glycosylation, of HDL-associated proteins on the immunomodulatory function of HDL.

Our study has several limitations. Firstly, this was a pilot study with a relatively small sample size in each clinical group, which may explain why some of our results did not reach statistical significance, and also requires further studies to corroborate our findings. We did not have many of the clinical measurements including LDL-C, TG, CRP for control subjects. In addition to larger sample size, future studies need to examine the effects of gender and age, in addition to other clinical characteristics (e.g. type 1 diabetes) and lifestyle factors (e.g. smoking) on HDL compositional and functional profiles. In this study we measured the secretion of IL-6 as a prototypical cytokine response in LPS-stimulated monocytes. However, future studies should examine a more comprehensive profile of cytokines, chemokines, adhesion molecules, and other inflammatory mediators, as well as other inflammatory stimuli and in additional immune cell types to gauge the impact of HDL composition on its ability to modulate immune response.

## Conclusions

We found that HDL glycoprotein and distinct site-specific glycosylation were characteristic of clinical groups, with large differences and characteristic glycopeptide profiles among diabetic patients undergoing HD, subjects with MetS and healthy controls. Generally, loss of sialic acid on an array of glycopeptides was associated with disease, except that in HD patients, HDL was enriched in di-sialylated ApoC-III isoform (ApoC-III_2_) and depleted in the less sialylated ApoC-III_1_ and ApoC-III_0_ isoforms. Yet specific glycans at specific sites did not follow these general patterns, highlighting the importance of comprehensive, site-specific glycoprofiling for biomarker discovery. Although, whether subjects were healthy or unhealthy strongly influenced their HDL glycoprofiles, the subjects’ clinical characteristics were not indicative of whether their HDL were pro- vs. anti-stimulatory. Instead, the glycoprofiles of key HDL-associated glycoproteins differentiated between those whose HDL induced vs. suppressed IL-6 secretion in stimulated monocytes. Finally, our data suggest that HDL glycoprotein profiles could potentially be predictive of susceptibility to serious infectious events. Together, these findings implicate glycoprofiling of HDL particles as a potential tool for developing biomarkers of disease, as well as for understanding the mechanisms that mediate the immunomodulatory function of HDL particles.

## Additional Information

**How to cite this article**: Krishnan, S. *et al*. HDL Glycoprotein Composition and Site-Specific Glycosylation Differentiates Between Clinical Groups and Affects IL-6 Secretion in *Lipopolysaccharide*-Stimulated Monocytes. *Sci. Rep.* **7**, 43728; doi: 10.1038/srep43728 (2017).

**Publisher's note:** Springer Nature remains neutral with regard to jurisdictional claims in published maps and institutional affiliations.

## Supplementary Material

Supplementary Information

## Figures and Tables

**Table 1:** Characteristics of the subjects within each group: healthy controls, diabetic patients on hemodialysis (HD), and subjects with metabolic syndrome (MetS).

	Control (n = 13)	HD (n = 24)	MetS (n = 13)
	Mean ± SD	Mean ± SD	Mean ± SD
BMI (kg/m2)	22.71 ± 2.96^a^	29.18 ± 5.92^b^	31.77 ± 3.30^b^
Systolic Blood pressure (mmHG)	114.39 ± 11.19^a^	151.33 ± 20.50^b^	126.12 ± 17.54^a^
Diastolic Blood pressure (mmHg)	72.83 ± 8.82	79.88 ± 11.78	76.96 ± 11.94
Age (y)	33.33 ± 10.21^a^	55.00 ± 13.45^b^	44.69 ± 13.31^a,b^
Weighted Average HDL size (nm)	14.65 ± 7.69	17.68 ± 9.26	11.41 ± 7.48
IL-6 (% response)	−6.07 ± 17.07	12.91 ± 66.56	−11.61 ± 25.42
CRP (mg/L)		19.83 ± 43.57	4.99 ± 3.88
TG (mg/dL)		115.83 ± 70.26^a^	174.24 ± 57.94^b^
**Composition of isolated HDL fraction**			
Cholesterol (mg/dL)	110.51 ± 42.83^a^	74.77 ± 24.61^b^	74.24 ± 25.00^a,b^
A1AT^^^	2.22 ± 1.72	3.43 ± 2.03	2.62 ± 1.99
A2HSG^^^	0.36 ± 0.17^a,b^	0.37 ± 0.17^a^	0.61 ± 0.38^b^
ApoC-III^^^	13.47 ± 3.43^a^	34.26 ± 16.25^b^	25.54 ± 11.77^b^
SAA^^^	0.04 ± 0.02^a^	7.74 ± 2.56^b^	0.17 ± 0.14^b^
LBP^^^	20.15 ± 8.85^a^	50.78 ± 24.70^b^	35.44 ± 15.55^b^
ApoA-I^^^	0.91 ± 0.39	0.91 ± 0.49	0.66 ± 0.26

Significant differences (‘a’ and ‘b’ superscripts indicate individual group differences at p < 0.05) using van der Weardan’s non parametric test, followed by Steel-Dwaas multiple comparison tests; ^^^Indicates that it was normalized to cholesterol (mg/dL) in the HDL fraction.

**Table 2:** Characteristics of the diabetic patients on hemodialysis who did not have an infectious event (HD−) vs. those who had an infectious hospitalization event within 60 days (HD+).

	HD(−) (n = 12)	HD(+) (n = 12)
	Mean ± SD	Mean ± SD
BMI (kg/m2)	28.95 ± 6.10	29.41 ± 6.00
Systolic Blood pressure (mmHG)	159.08 ± 14.74	143.58 ± 22.98
Diastolic Blood pressure (mmHg)	82.58 ± 11.11	77.17 ± 12.26
Age (y)	53.64 ± 14.99	56.88 ± 11.72
Weighted Average HDL size (nm)	21.98 ± 8.51	13.38 ± 8.14
IL-6 (% response)	−6.00 ± 29.04	31.83 ± 87.41
CRP (mg/L)	6.30 ± 5.13	19.83 ± 43.57
HDL-C (mg/dL)	57.38 ± 16.52	50.67 ± 13.16
TG (mg/dL)	109.46 ± 49.94	115.83 ± 70.26
**Composition of isolated HDL fraction**		
Cholesterol (mg/dL)	80.69 ± 19.68	68.84 ± 28.32
A1AT^	3.20 ± 1.55	3.65 ± 2.47
A2HSG^	0.32 ± 0.09	0.41 ± 0.21
ApoC-III^	30.85 ± 12.55	37.66 ± 19.20
SAA^	0.83 ± 2.21	4.28 ± 10.68
LBP^	44.45 ± 15.76	57.11 ± 30.65
ApoA-I^	0.90 ± 0.47	0.92 ± 0.53

^^^Indicates normalization to cholesterol (mg/dL) in the HDL fraction.
