# Supplementary material for: HDL Glycoprotein Composition and Site-Specific Glycosylation Differentiates Between Clinical Groups and Affects IL-6 Secretion in Lipopolysaccharide-Stimulated Monocytes
Source: Sci Rep. 2017 Mar 13;7:43728. doi: 10.1038/srep43728 (PMC5347119; doi:10.1038/srep43728)
Supplement: Supplementary Information [file srep43728-s1.doc]

**HDL Glycoprotein Composition and Site-Specific Glycosylation Differentiates Between Clinical Groups and Affects IL-6 Secretion in *Lipopolysaccharide*-Stimulated Monocytes**

Sridevi Krishnan, Michiko Shimoda, Romina Sacchi, Muchena J. Kailemia, Guillaume Luxardi, George A. Kaysen, Atul N. Parikh, Viviane N Ngassam, Kirsten Johansen, Glenn Chertow, Barbara Grimes, Jennifer T. Smilowitz, Emanual Maverakis, Carlito B. Lebrilla, Angela M. Zivkovic


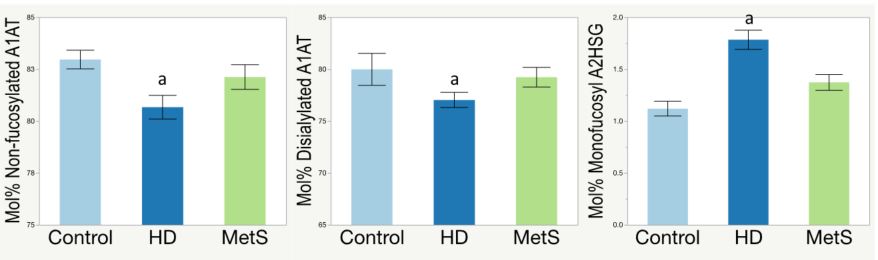


**Supplemental Figure S1.** Difference between clinical groups in non-fucosylated alpha-1 antitrypsin (A1AT), disialytated A1AT and monofucosylated alpha-2HS-glycoprotein (A2HSG) between control subjects, diabetic patients on hemodialysis (HD), and subjects with metabolic syndrome (MetS). Significant differences ('a' subscript indicate individual group differences at p<0.05).

**
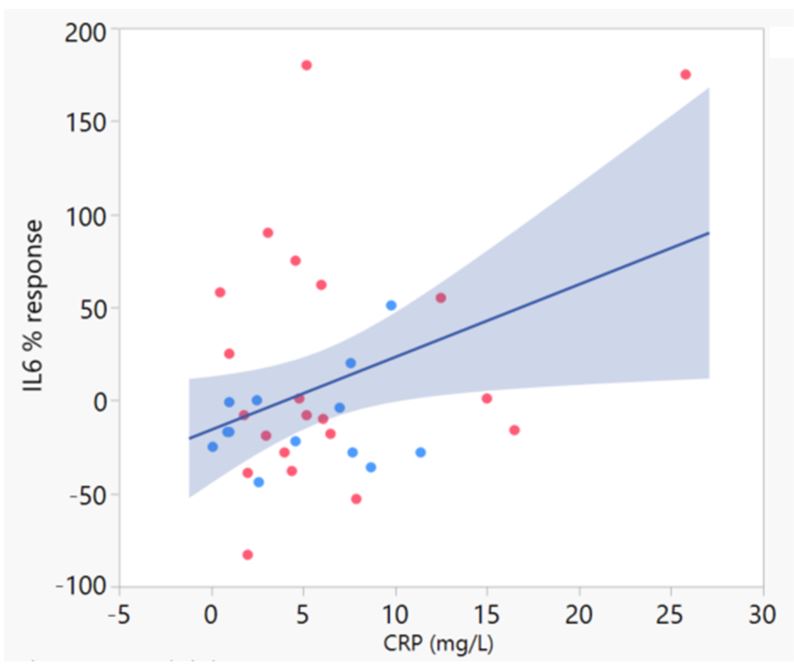
**

**Supplemental Figure S2**: Positive correlation between IL-6 percent response and CRP (mg/dL) in n=37 patients (diabetic patients on hemodialysis, red dots; and patients with metabolic syndrome, blue dots).
